# Comparisons of oblique lumbar interbody fusion and transforaminal lumbar interbody fusion for degenerative spondylolisthesis: a prospective cohort study with a 2-year follow-up

**DOI:** 10.3389/fsurg.2023.1115823

**Published:** 2023-04-27

**Authors:** Jingye Wu, Jintao Ao, Zhongning Xu, Guanqing Li, Tenghui Ge, Yongqing Wang, Xiaohui Tao, Wei Tian, Yuqing Sun

**Affiliations:** Beijing Jishuitan Hospital, Beijing, China

**Keywords:** interbody fusion, comparative study, OLIF, spondylolisthesis, cohort study

## Abstract

**Objective:**

This study aimed to compare the clinical outcomes between oblique (OLIF) and transforaminal lumbar interbody fusion (TLIF) for patients with degenerative spondylolisthesis during a 2-year follow-up.

**Methods:**

Patients with symptomatic degenerative spondylolisthesis who underwent OLIF (OLIF group) or TLIF (TLIF group) were prospectively enrolled in the authors’ hospital and followed up for 2 years. The primary outcomes were treatment effects [changes in visual analog score (VAS) and Oswestry disability index (ODI) from baseline] at 2 years after surgery; these were compared between two groups. Patient characteristics, radiographic parameters, fusion status, and complication rates were also compared.

**Results:**

In total, 45 patients were eligible for the OLIF group and 47 patients for the TLIF group. The rates of follow-up were 89% and 87% at 2 years, respectively. The comparisons of primary outcomes demonstrated no different changes in VAS-leg (OLIF, 3.4 vs. TLIF, 2.7), VAS-back (OLIF, 2.5 vs. TLIF, 2.1), and ODI (OLIF, 26.8 vs. TLIF, 30). The fusion rates were 86.1% in the TLIF group and 92.5% in the OLIF group at 2 years (*P *= 0.365). The OLIF group had less estimated blood loss (median, 200 ml) than the TLIF group (median, 300 ml) (*P* < 0.001). Greater restoration of disc height was obtained by OLIF (mean, 4.6 mm) than the TLIF group (mean, 1.3 mm) in the early postoperative period (*P* < 0.001). The subsidence rate was lower in the OLIF group than that in the TLIF group (17.5% vs. 38.9%, *P *= 0.037). The rates of total problematic complications were not different between the two groups (OLIF, 14.6% vs. TLIF, 26.2%, *P *= 0.192).

**Conclusion:**

OLIF did not show better clinical outcomes than TLIF for degenerative spondylolisthesis, except for lesser blood loss, greater disc height restoration, and lower subsidence rate.

## Introduction

As a minimally invasive approach, oblique lumbar interbody fusion (OLIF) was first introduced by Silvestre et al. in 2012 (1). For patients with lumbar spinal deformity requiring corrective surgery and multiple-level fusion, OLIF is superior to the conventional posterior lumbar fusion techniques, as better angular correction, less blood loss, and less severe surgical trauma were achieved by OLIF (2).

For degenerative spondylolisthesis, lumbar interbody fusion is one of the most common procedures, and different surgical approaches have been reported, including anterior, posterior, transforaminal (TLIF), and lateral (3). Through a muscle-splitting approach, OLIF allows for large-size cage insertion, producing indirect decompression by enlargement of the spinal canal and intervertebral foramen (4). The clinical outcomes in previous studies are effective for degenerative spondylolisthesis by OLIF with indirect decompression and short-level fusion (5, 6). However, whether OLIF is superior to the conventional TLIF for degenerative spondylolisthesis concerns many surgeons. Few comparative studies were retrospective and showed inconsistent results with short-term follow-ups (7–9).

This prospective study aimed to compare the clinical outcomes between OLIF and TLIF for patients with degenerative spondylolisthesis during a 2-year follow-up.

## Materials and methods

### Study design

This is a prospective cohort study comparing the treatment effect between two groups: patients who underwent OLIF or TLIF. The protocol of this study was approved by the ethical committee of the authors’ hospital, and informed content was obtained for all eligible patients.

The sample size was estimated to be 45 patients for each group, with 80% power to detect the between-group difference of 10 on the magnitude of Oswestry disability index (ODI) improvement at a two-sided significance level of 0.05. A difference of 16 ODI improvement between OLIF and TLIF groups, which was derived from previous studies (6, 10), an SD of 10 for the ODI improvement, and a rate of loss to 2-year follow-up of 20% were assumed.

### Patient population

Eligible patients who underwent OLIF were prospectively and consecutively enrolled from July 2017, and those who underwent TLIF were enrolled from January 2018 in the authors’ hospital. The inclusion criteria were symptomatic radiculopathy or claudication, which was disabling and intolerable for more than 3 months with failed conservative management, degenerative spondylolisthesis, Meyerding grade I or II slip, unstable slip evidenced by mechanical low back pain with excessive motion on flexion–extension lumbar radiographs, and planned single-level or two-level fusion. The fusion extending to the adjacent level with symptomatic spinal stenosis was also eligible. The exclusion criteria were lumbar scoliosis greater than 30°, concomitant infection, tumor or fresh fracture at the lumbar spine, previous lumbar surgery, coexistent pathology at hip or knee joint causing unremitting leg pain or severe disability, previous knee or hip joint replacement, and rheumatoid arthritis.

The choice of OLIF or TLIF depends on the surgeon's preference and the patient’s consent. All surgeons were experienced with at least 50 surgical cases for OLIF or TLIF they performed in this study.

### Procedures

OLIF was performed according to the Medtronic OLIF25 surgical technique. An appropriate size of 6° lordotic cage (18 mm in width, CLYDESDALE Spinal System, Medtronic) was inserted into proper position, which was confirmed by fluoroscopy. The bone grafts in the cage were allografts mixed with demineralized bone matrix (AlloMatrix, Wright Medical). Posterior fixation at the prone position was performed. Percutaneous pedicle screw fixation was performed if indirect neural decompression was appropriate for selected patients.

Patients with one of the following conditions underwent direct neural decompression and open fixation: preoperative radiating pain at bed rest, migrating disc or ossification at the spinal canal, and ankylosed facet joint. For these patients, partial laminectomy or laminotomy and pedicle screw placements were performed.

TLIF was performed in an open fashion at the prone position. Through the posterior midline approach, pedicle screws were inserted. Afterward, unilateral facetectomy, neural decompression, endplate preparation, and insertion of the PEEK cage with morselized autograft were performed. Patients with bilateral neurological symptoms underwent bilateral decompressions; otherwise, unilateral decompression was performed during TLIF procedures.

### Outcome measures

The pain intensity and severity of disability were measured using the self-reported visual analog score (VAS) and ODI. The primary outcomes are treatment effect (changes of VAS and ODI from baseline) at 2 years after surgery.

The enrolled patients were followed up at 3, 6, 12, and 24 months by two coordinators (TG and GL). VAS and ODI were collected through an online survey tool at each timepoint of follow-up. Patients returned to the authors’ hospital for radiographic assessment at 3, 12, and 24 months. Standing anteroposterior, lateral, and flexion–extension radiographs were obtained. CT scans were obtained at the last follow-up (2 years or more).

**Figure 1:**
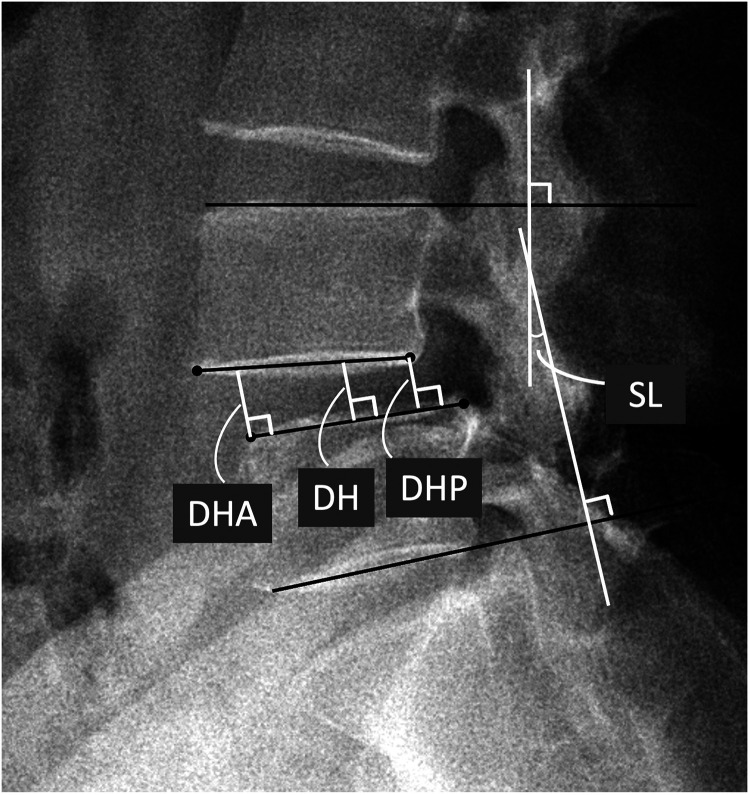
Definitions of radiographic parameters. SL, DH, DHA, and DHP on the standing lateral view of lumbar spine. SL, segmental lordosis; DH. disc height; DHA, anterior DH; DHP, posterior DH.

Radiographic parameters include disc height (DH), anterior DH (DHA), posterior DH (DHP), and segmental lordosis (SL) at the slip level (see Figure 1). These parameters were measured on the lateral standing radiographs before the operation, during the early postoperative period (within 3 days), and at the last follow-up.

The fusion status was assessed on flexion–extension radiographs and CT scans at the last follow-up. Less than 5° rotation and 3 mm displacement (indicating excessive motion) on flexion–extension radiographs and grade I or II bone bridging on CT scans were regarded as fusion; otherwise, failure of fusion was considered. The grading method of bone bridging was described by Isaacs et al. (11). Endplate injury was confirmed if endplate encroachment was greater than 2 mm on the early postoperative radiograph. Cage subsidence was measured on CT scans at the last follow-up using the grading method described by Marchi et al. (grade I–III, higher grade indicating severe subsidence) (12).

Two orthopedic surgeons (JA and ZX) were trained and measured radiographic parameters independently on Carestream PACS (version 11.0). Fusion status, endplate injury, and cage subsidence were also assessed by these surgeons. The agreement of the measurement was evaluated by an interclass correlation coefficient (>0.75 is acceptable). The mean value of the two observers’ results was used for statistical analysis. If grading inconsistency existed between two observers, a third observer (J.W) would ultimately confirm the grade.

The surgical complications for each group were evaluated. The complications comprised mechanical complications (fusion status, endplate injury, cage subsidence, or failure of fusion), neurological injury, visceral injury, surgical site infection, excessive bleeding, and death.

### Statistical analysis

Comparisons of continuous variables between two groups were performed by using an independent sample *t*-test if normal distributions were confirmed by the Shapiro–Wilk test. Otherwise, nonparametric analysis was performed by using the Mann–Whitney *U* test. For categorical variables, the differences between two groups were analyzed by a *χ*^2^ test.

The statistical analyses were performed using SPSS software, IBM SPSS Statistics, version 23.0. The statistically significant level of difference was assumed at *P* < 0.05 based on a two-sided hypothesis test.

## Results

**Figure 2:**
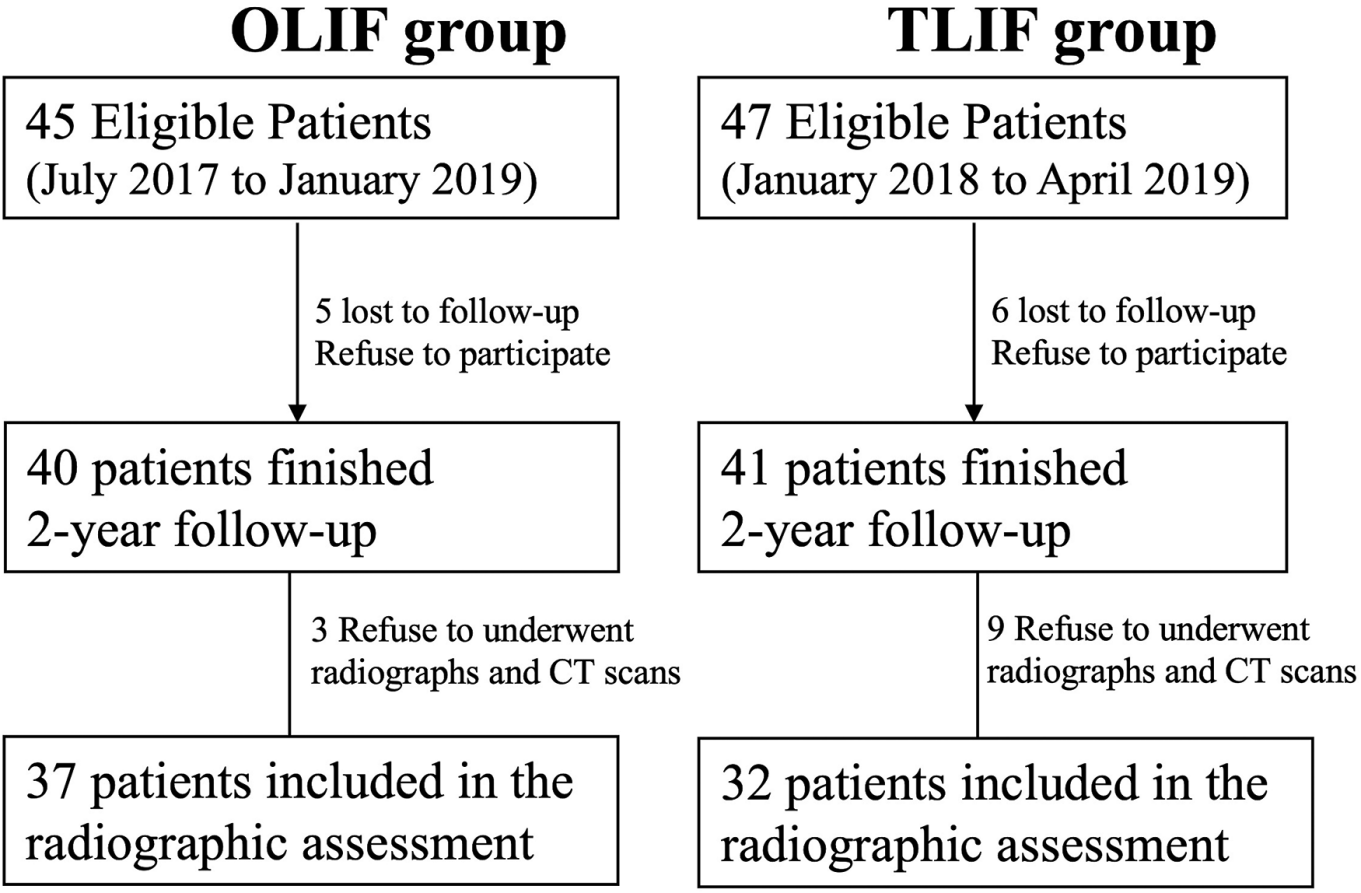
Enrollment and 2-year follow-up.

**Figure 3:**
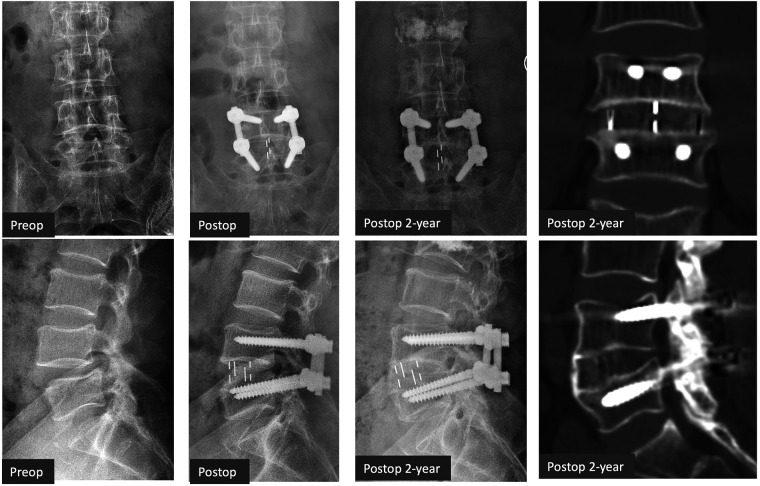
Case example of OLIF. A 63-year-old male patient with L4/5 degenerative spondylolisthesis had symptomatic low back pain and radiculopathy (ODI 35.6%, VAS-Leg 6, VAS-Back 6), which were relieved after OLIF with posterior laminotomy and fixation (ODI 10%, VAS-Leg 1, VAS-Back 2) at 2 years. Grade 1 fusion (apparent bone bridging) on CT scans was achieved at 2 years. OLIF, oblique lumbar interbody fusion; ODI, Oswestry disability index; VAS, visual analog scale.

**Figure 4:**
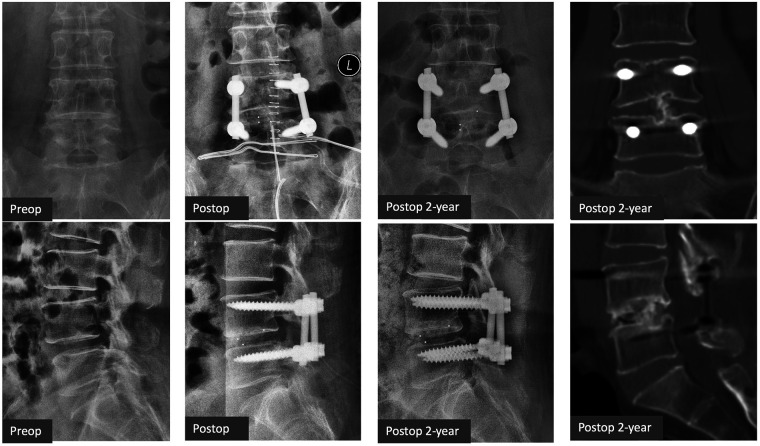
Case example of TLIF. A 56-year-old female patient with L4/5 degenerative spondylolisthesis had neurological claudication and low back pain (ODI 50%, VAS-Leg 5, VAS-Back 5). The symptoms were subsided after TLIF (ODI 16%, VAS-Leg 1, VAS-Back 2) at 2 years. Grade 2 fusion (patchy bone bridging) on CT scans was achieved at 2 years. TLIF, transforaminal lumbar interbody fusion; ODI, Oswestry disability index; VAS, visual analog scale.

A total of 45 patients were eligible for the OLIF group and 47 patients for the TLIF group. Forty patients finished the 2-year follow-up in the OLIF group and 41 patients in the TLIF group (the workflow of the follow-up is shown in Figure 2). The rates of follow-up were both greater than 80%. Case examples of OLIF and TLIF were shown on Figures 3, 4.

### Patient characteristics

Patient characteristics were shown in Table 1. Higher proportion of two-level fusion (51.2%) was found in TLIF group than did the OLIF group (27.5%). The duration of operation in OLIF group (mean, 190.3 min) was longer than those in TLIF group (mean, 157 min) (*P* = 0.001). OLIF group had less estimated blood loss (median, 200 ml) than did TLIF group (median, 300 ml) (*P* < 0.001). Thirteen cases had greater than 500 ml blood loss and two cases greater than 1,000 ml blood loss in TLIF group, whereas no OLIF cases producing greater than 500 ml blood loss. Patients in OLIF group stayed slightly shorter period in hospital postoperatively than those in TLIF group (*P* = 0.035).

**Table 1:** Patient characteristics in OLIF and TLIF groups^a^.

	TLIF	OLIF	*P*-value
Patients (*n*)	41	40	NA
Age (years)	61.2 ± 8.1	63.1 ± 8.2	0.311
Sex (female, %)	88%	75%	0.138
BMI (kg/m^2^)	25.9 ± 3.9	26.3 ± 3.7	0.576
Slippage level (*n*)	45	43	
L3/4	5	6	0.687
L4/5	40	37	
Single	37	37	0.718
Two-level	4	3	
Fused segments (*n*)			
1	20	29	**0** **.** **029**
2	21	11	
Duration of operation (min)	157.0 ± 43.1	190.3 ± 40.3	**0**.**001**
Estimated blood loss	300 (300)	200 (100)	**<0**.**001**
>500 ml (*n*)	13	0	**<0**.**001**
>1,000 ml (*n*)	2	0	
Postoperative hospital stay (days)	5.5 ± 1.3	4.9 ± 1.1	**0**.**035**
Duration of follow-up (days)	763 ± 69	783 ± 79	0.948

OLIF, oblique lumbar interbody fusion; TLIF, transforaminal lumbar interbody fusion.

Bold values mean *P*-value less than 0.05.

^a^ Data presented as means and SDs if normal distribution was met, otherwise as median (interquartile range).

### Primary outcomes

The comparisons of treatment effects at 2 years between the two groups demonstrated no difference for changes of VAS-leg (OLIF, 3.4 vs. TLIF, 2.7), VAS-back (OLIF, 2.5 vs. TLIF, 2.1), and ODI (OLIF, 26.8 vs. TLIF, 30). The results of comparisons during follow-up are shown in Table 2.

**Table 2:** Comparisons of VAS and ODI between OLIF (*n* = 40) and TLIF (*n* = 41) groups^a^.

	Preoperative			Postoperative 3-month			Postoperative month		
	OLIF	TLIF	*P*-value	OLIF	TLIF	*P*-value	OLIF	TLIF	*P*-value
VAS-back	4.8 ± 2.2	4.3 ± 1.6	0.289	3 (1)	2 (0)	0.003	3 (1)	2 (0)	**0.005**
VAS-leg	5.7 ± 1.9	4.9 ± 1.5	**0.041**	2 (1)	2 (0)	0.069	2 (1)	2 (1)	0.135
ODI	45.6 ± 16.6	45.7 ± 16.9	0.978	23.3 (13.1)	22 (14)	0.075	22 (15)	18 (13)	**0.041**
	Postoperative 1-year			Postoperative 2-year			Treatment effect at 2-year^b^		
	OLIF	TLIF	*P*-value	OLIF	TLIF	*P*-value	OLIF	TLIF	*P*-value
VAS-back	2 (1)	2 (0)	**0.024**	2 (1)	2 (1)	0.411	2.5 (1.8–3.2)	2.1 (1.6–2.6)	0.361
VAS-leg	2 (1)	2 (0)	0.218	2 (1)	2 (0)	0.226	3.4 (2.8–4.0)	2.7 (2.2–3.2)	0.095
ODI	20 (13)	18 (12.1)	0.099	19 (11.7)	14 (10)	0.069	26.8 (22–31.6)	30 (24.8–35.1)	0.367

OLIF, oblique lumbar interbody fusion; TLIF, transforaminal lumbar interbody fusion; VAS, visual analog scale; ODI, Oswestry disability index.

Bold values mean *P*-value less than 0.05.

^a^ Data presented as mean and SD if normal distribution was met, otherwise as median (interquartile range).

^b^ Treatment effect means the improvement of scores at 2 years, compared with preoperative scores. The values were presented with mean (95% confidential interval).

The comparisons of preoperative scores showed no significant difference, except the mean VAS-leg (OLIF, 5.7 ± 1.9 vs. TLIF, 4.9 ± 1.5, *P* = 0.041). During follow-up, TLIF group had slightly less back pain than did the OLIF group at 3 months, 6 months, and 1 year; however, the difference between groups became nonsignificant at 2 years (*P* = 0.411). The VAS-leg and ODI were similar between two groups during each follow-up, except ODI at 6 months (OLIF, median: 22 vs. TLIF, median: 18, *P* = 0.041).

Due to the heterogeneity in proportions of two-level fusion between groups, subgroup analysis was performed (Table 3). For one-level fusion, the OLIF group (mean, 3.6) had a greater reduction of leg pain at 2 years than did TLIF group (mean, 2.5) (*P* = 0.049), which was also clinically meaningful difference. For two-level fusion, however, no difference was found.

**Table 3:** Subgroup analysis of VAS and ODI between OLIF and TLIF groups^a^.

	Preoperative			Treatment effect at 2 years^b^		
	OLIF	TLIF	*P*-value	OLIF	TLIF	*P*-value
Single-level fusion comparison (TLIF 20 cases vs. OLIF 29 cases)						
VAS-back	4.9 ± 2.4	4.4 ± 1.8	0.618	2.6 (1.7–3.5)	2.1 (1.3–2.8)	0.351
VAS-leg	5.9 ± 1.8	4.8 ± 1.7	0.027	3.6 (2.9–4.3)	2.5 (1.7–3.4)	**0.049**
ODI	46.1 ± 17	43.7 ± 16.4	0.618	26.3 (20.9–31.7)	28.1 (20.8–35.3)	0.791
Two-level fusion comparison (TLIF 21 cases vs. OLIF 11 cases)						
VAS-back	4.5 ± 1.7	4.3 ± 1.4	0.829	2.2 (1.2–3.2)	2.2 (1.6–2.8)	0.987
VAS-leg	5.0 ± 2.1	5.0 ± 1.3	1.000	2.8 (1.4–4.3)	2.9 (2.4–3.5)	0.903
ODI	44.3 ± 16.1	47.6 ± 17.5	0.607	28.1 (16.2–39.9)	31.8 (23.9–39.6)	0.572

OLIF, oblique lumbar interbody fusion; TLIF, transforaminal lumbar interbody fusion; VAS, visual analog scale; ODI, Oswestry disability index.

Bold values mean *P*-value less than 0.05.

^a^ Data presented as mean and SD if normal distribution was met, otherwise as median (interquartile range).

^b^ Treatment effect means the improvement of scores at 2-year, compared with preoperative scores. The values were presented with mean (95% confidential interval).

### Radiographic evaluations

Thirty-seven patients in OLIF group and 32 patients in TLIF were available for radiographic evaluation at 2 years after surgery (Table 4). Preoperative radiographic parameters, comprising disc height and segmental lordosis, showed similar results between two groups. Greater restoration of disc height obtained by OLIF (mean, 4.6 mm) than did TLIF (mean, 1.3 mm) at early postoperative period. The restoration of disc height at central, anterior, or posterior in OLIF group remained greater than those in TLIF group at 2 years. However, OLIF did not show greater restoration of segmental lordosis, compared with TLIF, at neither early postoperative period nor 2 years.

**Table 4:** Comparisons of radiographic parameters between OLIF and TLIF groups^a^.

	Preoperative				Early postoperative (within 3 days)				Late postoperative (at 2 years)			
	DH	DHA	DHP	SL	DH	DHA	DHP	SL	DH	DHA	DHP	SL
TLIF	8.7 ± 1.9	10.6 ± 3.1	7.1 ± 1.9	12.9 ± 6.9	10.1 ± 1.8	12.4 ± 2.7	7.9 ± 1.9	14.4 ± 5.7	9.2 ± 2.1	11.2 ± 3.0	6.6 ± 1.8	12.7 ± 6.2
OLIF	8.3 ± 1.8	9.9 ± 2.5	6.8 ± 1.6	13.5 ± 7.6	13.0 ± 1.4	15.4 ± 2.2	9.9 ± 2.1	16.5 ± 7.3	11.8 ± 1.5	14.3 ± 2.3	8.4 ± 1.9	15.0 ± 7.4
*P*-value	0.378	0.270	0.460	0.732	**<0**.**001**	**<0**.**001**	**<0**.**001**	**0**.**154**	**<0**.**001**	**<0**.**001**	**<0**.**001**	0.137
	Changes from Preop^b^				Changes from Postop to 2-year follow-up^b^							
	DH	DHA	DHP	SL	DH	DHA	DHP	SL				
TLIF	1.3 ± 1.9	1.8 ± 2.5	0.8 ± 1.6	1.4 ± 5.4	0.8 ± 1.9	1.1 ± 2.3	1.2 ± 2.0	1.7 ± 3.7				
OLIF	4.6 ± 1.6	5.1 ± 2.4	3.1 ± 2.0	3.1 ± 5.5	1.2 ± 1.8	1.1 ± 2.5	1.4 ± 1.6	1.5 ± 2.9				
*P*-value	**<0**.**001**	**<0**.**001**	**<0**.**001**	0.209	0.471	0.910	0.551	0.807				

OLIF, oblique lumbar interbody fusion; TLIF, transforaminal lumbar interbody fusion; DH, disc height; DHA, anterior disc height; DHP, posterior disc height; SL, segmental lordosis.

Bold values mean *P*-value less than 0.05.

Totally 40 levels in OLIF group and 36 levels in TLIF were evaluated.

^a^ Data presented as mean and SD if normal distribution was met, otherwise as median (interquartile range).

^b^ Changes following operation means early postoperative values minus preoperative ones; changes following 2-year follow-up means late postoperative minus early postoperative ones.

Both DH parameters and SL had minimal loss in both groups during 2-year follow-up and the magnitude of DH or SL loss had no significant difference between two groups.

### Fusion status and complications

Fusion status and complications were shown in Table 5. Thirty-seven patients in OLIF group and 32 patients in TLIF were available for radiographic evaluation at 2 years after surgery. The fusion rates were 86.1% in TLIF group and 92.5% in OLIF group at 2 years, whereas there was no significant difference between two groups. For the implant-related complications, the rates of cage subsidence were different (TLIF, 38.9% vs. OLIF, 17.5%, *P* = 0.037). No differences were found in the rate of intraoperative endplate injury and vertebral fracture between two groups.

**Table 5:** Fusion status and total complications at 2-year follow-up.

	TLIF	OLIF	*P*-value
Mechanical failure			
Endplate injury	12 (33.3%)	5 (12.5%)	0.111
Vertebral fracture	0	2 (5%)	
Cage subsidence	14 (38.9%)	7 (17.5%)	**0**.**037**
Grade 1	11 (30.6%)	7 (17.5%)	
Grade 2	3 (8.3%)	0	
Non-fusion	5 (13.9%)	3 (7.5%)	
Class 1 (fused)	10	11	0.365
Class 2 (fused)	21	26	
Class 3 (not fused)	4	3	
Class 4 (not fused)	1	0	
Neurological injury			
Transient numbness or pain	0	6 (15%)	0.096
Permanent paresthesia	3 (7.3%)	2 (5%)	
Permanent motor deficit	0	0	
Others			
Excessive bleeding	2 (4.8%)		
Surgical site infection	1 (2.4%)		
Total problematic complications^a^	11 (26.2%)	5(12.5%)	0.118

OLIF, oblique lumbar interbody fusion; TLIF, transforaminal lumbar interbody fusion.

Bold values mean *P*-value less than 0.05.

^a^ Includes permanent nerve injury, excessive bleeding, non-fusion, surgical site infection.

For the rates of neurological injury, no differences were found. Three patients suffered from permanent nerve root injury in TLIF group, while three patients had paresthesia over groin area but normal hip flexion power due to permanent lumbar plexus injury in OLIF group. In addition, five patients reported transient lumbar plexus injury in OLIF group, which were relieved within 3 months.

Two patients suffered from excessive bleeding (>1 L) during TLIF and underwent blood transfusion. One surgical site infection occurred in TLIF group and subsequent reoperation for debridement and lavage were performed. The rates of problematic complications, which comprised permanent nerve injury, excessive bleeding, non-fusion, or surgical site infection, were not different between two groups (OLIF, 12.5% vs. TLIF, 26.2%, *P* = 0.118), although there was a trend that TLIF had more problematic complication rate.

## Discussion

Lower complication rates, less blood loss, and better corrective effect can be achieved by lateral approaches of interbody fusion for deformity-correction surgery, compared with conventional TLIF (13). For degenerative spondylolisthesis, however, the benefit of OLIF was debated (14). Similar clinical outcomes and complication rates were shown in this prospective comparative study, although less blood loss, shorter hospital stay, higher disc height, and lower subsidence rates were shown in OLIF group than in TLIF group.

### Clinical outcomes

The baseline characteristics were equivalent between two groups except for the proportion of two-level fusion and preoperative VAS-leg. The difference of VAS-leg between groups was only 0.8, not reaching clinically meaningful difference, which was regarded as comparable for two groups. Age (mean, 62.2 years), female proportion (82%), BMI (mean, 26.1 kg/m^2^), preoperative ODI (mean, 45.6), VAS-back (mean, 4.5), and VAS-leg (mean, 5.3) for all patients with degenerative spondylolisthesis were similar to the baseline characteristics of previous studies (15–17).

As primary outcomes, the changes of VAS-back (OLIF, 2.5 vs. TLIF, 2.1), VAS-leg (OLIF, 3.4 vs. TLIF, 2.7), and ODI (OLIF, 26.8 vs. TLIF, 30) were similar between two groups at 2 years postoperatively, which suggested equivalent treatment effect on pain relief and functional improvements by these two different approaches of interbody fusion. OLIF did not show better clinical outcomes than did TLIF for single-level or two-level degenerative spondylolisthesis. These nonsignificant difference on treatment effect were consistent with two previous retrospective studies of 1-year follow-up (8, 9). Although the VAS and ODI in both groups had statistically significant differences at 3-month, 6-month, and 1-year follow-up; however, the differences did not reach the clinically significant difference (18).

Given that TLIF group had higher proportion of two-level fusion (51.2% vs. 27.5% in OLIF group), subgroup analyses were performed and showed similar results. Only changes VAS-leg at 2-year revealed significant difference (OLIF, 3.6 vs. TLIF, 2.5) in single-level subgroup comparison, whereas the preoperative VAS-leg was not equal (OLIF: 5.9 vs. TLIF: 4.8), indicating the greater treatment effect of VAS-leg in OLIF group was created by greater preoperative VAS-leg.

### Fusion status and complications

Cage subsidence occurred at seven levels in OLIF group (17.5%), which were all Grade 1 (25%–50% subsidence). Previous study of lateral lumbar interbody fusion reported similar cage subsidence rate of 10% at 1-year follow-up by Malham et al. (19). In contrast, 14 levels (38.9%) in TLIF group had cage subsidence among those three levels had Grade 2 (50%–75% subsidence), which was close to 34.1% occurrence in the study by Yao et al. (20). Lower cage subsidence rate in OLIF group suggested anteriorly placed large-size cages with posterior fixation provide more stable construct than those in TLIF group.

The fusion rates of two groups in this study were compatible with previous systematic reviews (OLIF, 90.1% vs. TLIF, 87.1%) (19, 21). Allograft with demineralized bone matrix together with large-size cages were implanted in OLIF group, compared with morselized cancellous bone graft in bullet-shaped cages in TLIF group. With less cage subsidence, however, OLIF group did not have significantly higher fusion rate than did TLIF group (OLIF, 92.5% vs. TLIF, 86.1%), although the trend of higher fusion rate existed.

Abe et al. (22) reported 13.5% of 155 patients who underwent OLIF presented with transient neurological deficit, whereas only 1.2% permanent. In this study, six patients (12.5%) in OLIF group had transient paresthesia over groin area or psoas weakness, which were subsided until three months postoperatively. However, two patients (5%) had permanent paresthesia over groin area without motor deficit, which had relatively higher occurrence than did another study (reporting 2.6% permanent paresthesia) (23). Higher rate of paresthesia over groin area in this study probably caused by manipulation of the anterior portion of psoas (Zone 1), resulting in genitofemoral nerve injury (24). In contrast, three patients (7.3%) suffered from permanent paresthesia over lower limb without motor deficit in TLIF group.

The total problematic complications rates were similar between two groups, although there was a trend of lower complication rate in OLIF group (12.5% vs. TLIF, 26.2%). In TLIF group, unexpected excessive blood loss (>1,000 ml) occurred in two patients and surgical site infection occurred once.

### Radiographic parameters

Large-size cage with lordotic angle was used in OLIF, which greatly enlarge the intervertebral space with respect of disc height and segmental lordosis. Previous studies have proved the correction effect by lateral approach of interbody fusion, especially for spinal deformities. In this study, OLIF showed better improvement of disc height than did TLIF (OLIF, 4.6 mm vs. TLIF, 1.3 mm). Consistence results between two groups were also shown for disc height in previous retrospective comparative studies (7–9).

However, the improvement segmental lordosis between two groups showed inconsistent results, compared with previous studies. In this study, the improvement segmental lordosis was similar between two groups (OLIF: 3.1° vs. TLIF: 1.4°), similar to 2.8° changes in previous study of Lateral Lumbar Interbody Fusion (LLIF) (25). For degenerative spondylolisthesis, the local deformity is not severe. With slight loss of preoperative segmental lordosis (mean preoperative SL: 13.2°), the magnitude of angular correction by OLIF was limited.

### Limitations

Heterogeneity occurred in the comparison between two groups. The different proportions of two-level fusion existed, and the following subgroup analysis showed similar results to the whole group comparisons. However, the sample size in subgroups was decreased, which probably impaired the power of statistics to detect the group difference. In this study, heterogeneity also exited in techniques of OLIF. Half of patients underwent OLIF with direct decompression which means partial laminectomy through posterior midline approach. Hence, subgroup of OLIF without direct decompression (minimally invasive technique) was compared with TLIF group. Similarly, no differences were found in terms of pain relief and functional improvement.

This study was conducted in a single center. Although techniques of procedure and patient characteristics were similar to previous studies, differences may exist and may impact on the external validity of the result of this study.

## Data Availability

The raw data supporting the conclusions of this article will be made available by the authors, without undue reservation.
